# Evaluation of *Lactiplantibacillus plantarum* CNPC001 in Co-Culture with *Streptococcus thermophilus* QGE on Microbial Sanitary Indicators in Fermented Raw Goat Milk

**DOI:** 10.3390/microorganisms14040799

**Published:** 2026-04-01

**Authors:** Miqueas Oliveira Morais da Silva, Vanderlania do Nascimento Santos, Ana Paula Albuquerque da Silva, Beatriz Patrício Rocha, Giordanni Cabral Dantas, Isanna Menezes Florêncio, Elainy Virgínia dos Santos Pereira, Isadora Kaline Camelo Pires de Oliveira Galdino, Eliane Rolim Florentino, Samuel Carneiro de Barcelos, Karina Maria Olbrich dos Santos, Antônio Silvio do Egito, Flávia Carolina Alonso Buriti

**Affiliations:** 1 Programa de Pós-Graduação em Ciências Farmacêutica, Universidade Estadual da Paraíba, Campina Grande 58429-500, PB, Brazil; miqueas_morais@hotmail.com (M.O.M.d.S.);; 2 Núcleo de Pesquisa e Extensão em Alimentos, Universidade Estadual da Paraíba, Campina Grande 58429-500, PB, Brazil; 3 Centro de Ciências Biológicas e da Saúde, Universidade Estadual da Paraíba, Campina Grande 58429-500, PB, Brazil; 4 Programa de Pós-Graduação em Biotecnologia, Universidade Estadual do Ceará, Fortaliza 60714-903, CE, Brazil; 5 Embrapa Agroindústria de Alimentos, Av. das Américas, 29501, Rio de Janeiro 23020-470, RJ, Brazil; 6 Embrapa Caprinos e Ovinos, Núcleo Regional Nordeste, Empresa Brasileira de Pesquisa Agropecuária, Campina Grande 558428-095, PB, Brazil; antoniosilvio.egito@embrapa.br

**Keywords:** biopreservative effect, goat dairy product, native culture

## Abstract

The antimicrobial potential of the strain *Lactiplantibacillus plantarum* CNPC001 in fermented raw goat milk against contamination indicators (*Escherichia coli*, *Staphylococcus* spp., *Salmonella* spp., as well as yeasts and molds) was assessed. To do this, two distinct treatments were produced in triplicate in raw goat milk, one only with the starter *Streptococcus thermophilus* QGE (T1) and the other with the starter and *Lp. plantarum* CNPC001 in co-culture (T2). The main bio-preservative effect of *Lp. plantarum* CNPC001 in co-culture with *S. thermophilus* was verified against *E. coli* and *Staphylococcus* spp., in which *E. coli* was completely inhibited at the end of fermentation and *Staphylococcus* spp. remained below the method threshold (<2.00 log CFU/g) from the 14th day up to the end of storage. For *Salmonella* spp., a significant difference between the fermented milks was verified at the end of fermentation process, in which the absence of this microorganism was only verified in the T2. No significant differences between the T1 and T2 were verified for yeast and molds. The viability of *Lp. plantarum* remained above 7 log CFU g^−1^ for 28 days of storage. Therefore *Lp. plantarum* CNPC001 in co-culture with *S. thermophilus* QGE was able to demonstrate a bio-preservative effect in fermented raw goat milk, inhibiting the growth of *E. coli*, *S. aureus*, and *Salmonella* spp.

## 1. Introduction

The *Lactobacillaceae* family is composed of Gram-positive rod-shaped bacteria, facultative anaerobes, and belongs to the group of lactic acid bacteria, since lactic acid is the main end product of carbohydrate metabolism. This family is naturally found in the gastrointestinal tract (GIT) of humans and animals [1,2].

These microorganisms are of particular interest because they exhibit health-related properties, in addition to having a long history of use in food fermentations [3]. Studies indicate that most strains belonging to this family exhibit probiotic properties, which can be characterized by their ability to adhere to the intestinal epithelium and inhibit the proliferation of pathogens in the GIT through competitive mechanisms. Adhesion to the intestinal mucus is a desirable characteristic in probiotic bacteria, as it increases their persistence in the GIT and their ability to effectively colonize the intestine [4,5,6]. In addition, the incorporation of probiotic lactic cultures with proven antimicrobial activity may contribute to maintaining the microbiological quality of dairy products during storage [7].

The use of *Lactiplantibacillus plantarum* in foods has been confirmed as safe, being included on the “Qualified Presumption of Safety” list [8]. Studies have shown that *Lp. plantarum* exhibits good compatibility when used in the fermentation of dairy products, and its antibacterial properties are of interest for food safety, particularly in biopreservation technology [5,9].

Biopreservation extends the shelf life of foods and improves their safety [10,11]. The ability of lactic acid bacteria to inhibit the growth of pathogenic bacteria has been recognized for centuries; for this reason, they play an important role in preventing food spoilage [10,11,12]. The capacity to alter the composition of the intestinal microbiota and to inhibit the colonization or growth of pathogenic microorganisms is a hallmark characteristic of lactic acid bacteria species used as probiotics [12].

Several mechanisms can explain biopreservative activity, the main ones being competition for nutrients and adhesion sites; induction of changes in environmental conditions that are unfavorable to pathogenic bacteria; production of antimicrobial compounds; and, finally, modulation of the host immune responses [13]. Among lactic acid bacteria, *Lp. plantarum* is one of the main species, widely distributed due to its characteristics, particularly its functional properties and its role as a biopreservative in dairy products and fermented foods [14]. A previous study of our research group has shown the in vitro antimicrobial potential of some indigenous strains belonging to the collection of the Brazilian Agriculture Research Corporation (EMBRAPA), among those *Lp. plantarum* CNPC001 [15].

Milk that has not been heated beyond 40 °C or undergone any treatment that has an equivalent effect is defined as raw milk [16]. It is a medium containing high microbial diversity, including *Lactococcus, Lactobacillus*, *Pseudomonas*, *Micrococcus*, *Staphylococcus*, *Leuconostoc*, *Enterococcus*, *Streptococcus*, *Bacillus*, *Clostridium*, *Listeria*, *Acinetobacter*, *Alcaligenes*, *Flavobacterium*, and *Aeromonas*, as well as *Enterobacteriaceae* and yeasts [17]. Components that are foreign to milk but enter the milk via the udder or during or after milking are often detrimental to its quality [18]. According to Lucey [19], even raw milk from clinically health animals that appears to be of good quality (i.e., low total bacteria count) may contain pathogens. The prevalence of pathogens in raw milk is influenced by numerous factors, including farm size, number of animals on the farm, hygiene, farm management practices, milking facilities, season, and others [19]. In some tropical countries, high temperatures and poor hygienic standards prevail [18]. In this context, raw milk constitutes a broad environment for pathogenic and spoilage microorganisms [17,19].

In Brazil, the Paraíba State is the leading goat milk producer, with 23.9% of total revenue of the goat milk sector in the country [20,21]. Data on quality of raw goat milk in semi-arid region of Paraíba indicate the need to decrease microbial contamination [22,23]. In view of the study of the biopreservative potential of a native strain of *Lp. plantarum*, this capacity could be better understood using this microorganism in fermented raw goat milk in co-culture with a species traditionally used as a starter in dairy products, since another previous study of our research group showed that the use of *Lp. plantarum* as a sole culture was not enough to achieve the desirable acidification for fermented milks [5].

## 2. Materials and Methods

**Figure 1:**
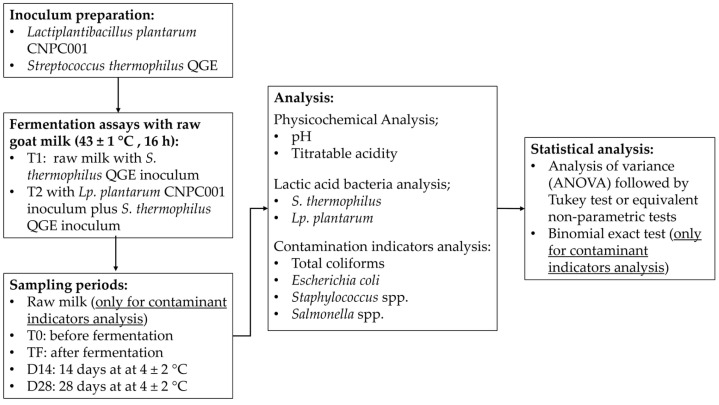
Main steps of the study methodology.

The main steps of the methodology of the present study are presented in Figure 1. These steps are fully detailed throughout Section 2.1, Section 2.2, Section 2.3, Section 2.4, Section 2.5, Section 2.6 and Section 2.7.

### 2.1. Inoculum Preparation

The native strain *Lp. plantarum* CNPC001 was provided by Embrapa Goats and Sheep, Sobral, CE, Brazil, and the commercial starter culture *S. thermophilus* QGE was provided by Biotech Brazil Fermentos e Coagulantes Ltda (Alto Piquiri, PR, Brazil). Both strains were supplied in lyophilized form. The following procedures were used to activate the strains. One loopful of the lyophilized *Lp. plantarum* CNPC001 culture was inoculated into sterile test tubes containing 10 mL of MRS broth (Acumedia, Neogen do Brasil para Laboratório, Indaiatuba, SP, Brazil). The tubes were then incubated in a bacteriological incubator (model 402–4D, Nova Ética, Vargem Grande Paulista, SP, Brazil) at 35 ± 2 °C for 48 h. Subsequently, the broth was distributed into 1.5 mL microtubes, and the bacterial culture was harvested by centrifugation at 3.0182× *g* for 10 min using a centrifuge (model CT-0603, PARSEC—Tecnologia Laboratorial do Brasil, Itajaí, SC, Brazil). The resulting pellet was carefully washed with 10 mL of 0.9% saline solution and centrifuged again under the same conditions to remove the MRS broth; this process was repeated three times. Finally, the pellet was inoculated into the dairy base to be fermented, thus originating the inoculum. This procedure was carried out separately for each strain.

Raw goat milk (300 mL) underwent a heat treatment (90 °C for 15 min) in a water bath and was subsequently cooled to 43 ± 1 °C. At this stage, three pellets of the native culture from the Embrapa collection of Microorganisms of Interest to the Food Industry and Agroenergy—Embrapa Food Technology (Rio de Janeiro, RJ, Brazil), *Lp. plantarum* CNPC001, were added. The milk was maintained at 43 ± 1 °C for 16 h, which was sufficient to reach a pH of approximately 5.0. Subsequently, the temperature of the dairy product was reduced to 4 ± 2 °C.

For the starter culture (*S. thermophilus*), raw goat milk underwent the same heat treatment described previously. After treatment, once the temperature reached 43 ± 1 °C, 0.004 g of the starter culture *S. thermophilus* was added per 100 g of goat milk. The milk was maintained at 43 ± 1 °C for 16 h, which was sufficient to reach a pH of approximately 5.0 and to improve consistency. Subsequently, the temperature of the dairy product was reduced to 4 ± 2 °C.

Thus, the initial inocula for both cultures were obtained and stored under refrigeration. These were continuously subcultured in thermally treated goat milk every 30 days. For each subculture, 5 g of the initial inoculum was used for 100 g of thermally treated goat milk.

### 2.2. Fermentation Assays with Raw Goat Milk

Two trials (Table 1) were defined: T1, obtained by adding the starter culture inoculum of *S. thermophilus* to raw milk, and T2, consisting of raw milk supplemented with inocula containing both the starter culture and the native culture *Lp. plantarum* CNPC001. Three batches (three independent biological replicates) were produced for each trial, without thermal treatment. Table 1 presents the ingredients used for the preparation of trials T1 and T2.

After preparation, the samples were placed in previously sanitized plastic bottles with caps (high density polyethylene, 150 mL) and labeled according to the sampling time periods. The samples were then fermented at 43 ± 1 °C for 16 h. At the end of the fermentation period, the temperature of the samples was reduced to 4 ± 2 °C. The samples were maintained in this temperature up to 28 days.

### 2.3. Sampling Time Periods

During fermentation, T0 corresponded to the time before fermentation, TF to the analysis after 16 h of fermentation, and additional analyses were performed after 14 (D14) and 28 days (D28) of storage under refrigeration at 4 ± 2 °C.

### 2.4. Physicochemical Analyses

The pH was determined using a potentiometer (Tecnal—Equipamentos para Laboratório Ltda., Piracicaba, SP, Brazil, model TEC-5), according to the analytical procedures described in method 017/IV of the Adolfo Lutz Institute [24], at times T0, TF, D14, and D28.

Titratable acidity of the samples was evaluated following the procedures described in method 426/IV of the Adolfo Lutz Institute [24] and expressed as g of lactic acid per 100 mL^−1^ at times T0, TF, D14, and D28.

### 2.5. Lactic Acid Bacteria Analyses

Microbiological analyses were performed on the triplicate dairy products before and after fermentation and after 14 and 28 days of storage by aseptically transferring 1.0 mL of the sample to 9.0 mL of sterile buffered peptone water (1 g L^−1^) and subjecting it to serial dilutions using the same diluent [7].

The *Lactobacillaceae* population was evaluated after growth on MRS agar acidified with acetic acid to pH 5.4 [5,7,9]. For this purpose, 1 mL aliquots of each dilution from the treatments were transferred, in duplicate, to sterile Petri dishes. Acidified MRS agar (adjusted to pH 5.4 with acetic acid), melted and cooled to approximately 45 °C, was then added. After homogenization and solidification of the agar, the plates were incubated at 35 ± 2 °C for 48 h under aerobic conditions.

Populations of *S. thermophilus* were determined by transferring 1 mL of each dilution, in duplicate, onto M17 agar (Difco, Sparks, MD, USA) supplemented with lactose (Vetec, Duque de Caxias, RJ, Brazil, 5 g L^−1^), followed by incubation at 35 ± 2 °C for 48 h [5,7,9].

### 2.6. Analysis of Contamination Indicators in Raw Milk and Raw Milk Supplemented with Lactic Acid Bacteria

Microbiological analyses were performed on the triplicate dairy products at the previously described sampling times (T0, TF, D14, and D28) and on raw milk by aseptically transferring 1.0 mL of the sample to 9.0 mL of sterile buffered peptone water (1 g L^−1^), followed by serial dilutions using the same diluent [7].

To determine the presence of *Staphylococcus* spp., 100 µL from the dilutions were surface-plated in duplicates onto Petri dishes containing mannitol salt agar (Kasvi). The plates were incubated at 35 ± 2 °C for 48 h [25].

The determination of total coliforms and *E. coli* was performed according to AOAC method 991.14 [26]. Aliquots of each sample dilution were transferred, in duplicate, onto Petrifilm™ EC plates (3M Microbiology, St. Paul, MN, USA) for the enumeration of coliforms and *Escherichia coli*. The Petrifilm™ EC plates were incubated at 35 ± 2 °C for 24 h. The presence of *E. coli* was confirmed on plates showing blue colonies with gas formation. For total coliforms, the sum of pink and blue colonies with gas production was considered.

The enumeration of molds and yeasts was carried out according to the methodology of Karagöz and Demirdöven [27] using potato dextrose agar (Himedia, Mumbai, India) acidified with tartaric acid to pH 3.5. Aliquots of 100 µL from serial dilutions were inoculated in duplicates onto the plates, which were stored without inversion at 25 ± 1 °C for 7 days, followed by colony counting.

For the detection of *Salmonella* spp., 25 g portions of each trial (aseptically collected) were homogenized with 225 mL of 0.1% peptone water (10^−1^ dilution) and incubated at 35 ± 2 °C for 24 h to obtain the 10^−1^ enrichment. Using a bacteriological loop, an aliquot of the enriched sample was streaked in duplicates onto Petri dishes containing RajHans differential medium for *Salmonella* spp. (Himedia) [28].

### 2.7. Statistical Analysis of the Results

Except for contamination indicator data, which were reported as ranges of minimum and maximum values, the results are presented as mean ± standard deviation. Statistical analyses were performed using the Statistica 6.0 software (Statsoft Inc., Tulsa, OK, USA). The results were first evaluated for normality using the Shapiro–Wilk test and for homogeneity of variances using Bartlett’s test.

When normality and/or homogeneity of variances were confirmed, the data were analyzed by analysis of variance (ANOVA), considering a significance level of α = 0.05, with Tukey’s test used to evaluate contrasts. When normality and/or homogeneity of variances were not confirmed, the data were analyzed using equivalent nonparametric tests, assuming a significance level of α = 0.05.

Statistical analysis of contamination indicator results was performed using the exact binomial test [29], adopting a significance level of α = 0.05. The null hypothesis for coliforms, *E. coli*, *Staphylococcus* spp., and molds and yeasts consisted of achieving an inhibitory effect of at least 33.33% in relation to the number of assays in which these microorganisms were detected. For *Salmonella* spp., the null hypothesis considered inhibition until the absence of this microorganism in the assays performed.

## 3. Results and Discussion

### 3.1. Physicochemical and Microbiological Parameters

Treatments T1 and T2, produced from fermented raw milk, were analyzed in triplicate. Table 2 presents the results of titratable acidity and pH obtained in the evaluated assays (raw milk supplemented with a native strain candidate for probiotic use, in the presence or absence of *Streptococcus thermophilus*).

The pH values recorded in the assays prior to the incubation process showed slight but significant differences (*p* ≤ 0.05) between trials, remaining close to 6.4. This low pH and significant differences between trials before fermentation are probable related to their microbiological profile [30]. After 16 h (end of fermentation), pH decreased significantly (*p* ≤ 0.05). The progressive fermentation of lactose due to the active metabolism of the added cultures may result in pH reduction. The treatments showed significant differences (*p* ≤ 0.05) in titratable acidity throughout storage, but no significant differences were observed between treatments on the same storage day. In both treatments, acidity values higher than 0.6 g of lactic acid 100 g^−1^ were achieved after 16 h of fermentation.

Milk acidification is also responsible for the texture and aroma of fermented milk due to the formation of organic acids, such as lactic acid, during fermentation. In agreement with these results, Galdino et al. [5] reported acidity values higher than 0.6 g of lactic acid 100 g^−1^ after 24 h of fermentation in a trial containing *Lp. plantarum* CNPC001 and *S. thermophilus*. According to Dimitrellou et al. [31], goat milk reaches higher acidity levels than cow milk when fermented. In the study of Serhan et al. [32], the increase in goat milk in the fermented milks tended to reduce the pH and protein content with an increase in total lactic acid bacteria, moisture, fat, and ash (therefore reducing lactose content). According to the authors, this could be a result of an increased peptidase activity of the lactic acid bacteria used in goat milk fermentation.

### 3.2. Evaluation of Lactic Acid Bacteria in Fermented Raw Milk

The viability of the starter *Streptococcus thermophilus* during processing and storage of the fermented goat dairy product is presented in Table 3. The populations of this culture initially showed no significant differences between treatments T1 and T2. In both treatments, the starter culture increased significantly by the end of the storage period. The populations of *S. thermophilus* differed significantly during storage (*p* ≤ 0.05) within the same treatment.

After 16 h fermentation (TF), populations of *S. thermophilus* in T2 trial with *Lp. plantarum* were statistically significantly higher than those verified in T1 (*p* ≤ 0.05), although biologically, this increase was lower than 0.2 log cycle. At the end of the experiment, the population of *S. thermophilus* remained above 7.9 log CFU g^−1^ for both treatments.

In contrast, a study conducted by Tian et al. [33] reported that *S. thermophilus* showed lower growth capacity during fermentation and reduced viability throughout the storage of cow’s milk yogurt when co-cultured with the probiotics *Lacticaseibacillus casei* LC2W (formerly *Lactobacillus casei* LC2W), *Lp. plantarum* ST-III, or *L. rhamnosus* GG, compared to fermentation without these probiotics.

Trial T2 showed *Lp. plantarum* counts above 6 log CFU g^−1^ (Table 4) from the first sampling point, with a significant increase (*p* ≤ 0.05) throughout storage. Thus, the native culture remained viable during the entire storage period and may therefore contribute a bioconservative effect in raw milk for at least 28 days of shelf life.

When evaluating the fermentative behavior of the potentially probiotic autochthonous culture *Lp. plantarum* CNPC001 in goat milk after thermal treatment, Galdino et al. [5] observed, from the initial fermentation time (0 h) to 6 h, 24 h, and 48 h of fermentation, that counts of both *Lp. plantarum* (8.10 ± 0.23, 8.42 ± 0.15, 7.85 ± 0.36, and 5.90 ± 0.12) and *Streptococcus thermophilus* (8.22 ± 0.39, 8.63 ± 0.30, 8.05 ± 0.04, and 5.99 ± 0.27, respectively) increased after 6 h and decreased after 24 h. In the present study, a significant increase in *S. thermophilus* (Table 3) and *Lp. plantarum* (Table 4) was observed between the initial fermentation time (T0) and after 16 h of fermentation (TF) for all samples. Initial higher counts in the study of Galdino et al. [5] were possibly related to the form of addition of lactic cultures in the products, since pellet with cells of *Lp. plantarum* (grow in MRS broth) and direct vat set lyophilized culture of *S. thermophilus* were added (they were not previously grown in milk for use of portions of cultured milk as inocula).

### 3.3. Evaluation of Contamination Indicators

Studies have demonstrated that strains from the family *Lactobacillaceae*, used alone or in co-culture with other lactic acid bacteria, inhibit the growth of pathogenic and spoilage microorganisms, including coliforms, *Escherichia coli*, *Staphylococcus aureus*, and *Salmonella* spp. [7,34,35,36].

In Table 5, reductions in the percentage (%) of either positive samples or samples above the legal limit to values of 33.0% or lower were considered significant. This reduction level was achieved for *E. coli* and *Staphhylococcus* spp. in raw milk fermented with *Lp. plantarum* in association with *S. thermophilus* (T2).

Moreover, a marked reduction in the microbial load of contaminants was observed in the T2 trial at the end of the storage period, compared with the unfermented raw milk (Table 5). For coliforms, although no significant differences were observed between treatments T1 and T2, on the 28th day of storage, only T2 complied with the limits established by Brazilian legislation for fermented milks [37].

Regarding *E. coli* populations (Table 5), a significant reduction in counts was observed after 16 h of fermentation (TF) for both treatments (T1 and T2). The percentage of positive samples and samples exceeding the legal limits in T2 (25.0%) was 50.0% lower than that observed for T1 (50.0%). Thus, the association of *Lp. plantarum* and *S. thermophilus* in fermented raw milk showed better bioconservative performance against *E. coli*.

Similarly to that observed for the inhibition of *E. coli* and *Staphylococcus* spp. by *S. thermophilus* in co-culture with *Lp. plantarum* in fermented goat milk in the present study, Goranov et al. [22], who intentionally contaminated samples with *E. coli* ATCC 25922 and *S. aureus* ATCC 25923, reported that *Lactobacillus helveticus* 2/20, either in free or encapsulated form, effectively inhibited the growth of these microbial indicators in chocolate mousse stored at 20 ± 2 °C.

Considering all samples analyzed over the four sampling periods, trial T1 (Table 5) produced positive samples for *Staphylococcus* spp. throughout the entire storage period (100.0%). In contrast, trial T2 (Table 5) showed 33.33% positive samples for *Staphylococcus* spp., and by the 14th day of storage, the results were below 2 log mL^−1^, indicating an inhibitory effect of the native culture *Lp. plantarum* in fermented raw milk. Consistent with these findings, it has been reported in vitro that *Lp. plantarum* TW29-1 exhibited strong antibacterial activity against *E. coli*, *S. aureus*, and *Salmonella* spp. [35].

Buriti, Cardarelli, and Saad [7] investigated the bioconserving potential of a co-culture of *Lacticaseibacillus paracasei* LBC 82 (formerly *Lactobacillus paracasei* LBC 82) and *Streptococcus thermophilus* in fresh cream cheese. Their findings indicated inhibition of bacterial contaminants from the beginning of manufacture and throughout 21 days of storage.

Milioni et al. [36] evaluated *Lactiplantibacillus plantarum* LpU4 and its purified plantaricin LpU4 as bioconservants and observed effective inhibition of *Staphylococcus aureus*, supporting the use of *Lp. plantarum* against this pathogen. No significant reduction in molds and yeasts was observed in any treatment; both produced positive samples exceeding legal limits across all four sampling periods. However, in T2, mold and yeast counts remained between 3.0 and 3.67 log CFU mL^−1^ throughout storage, suggesting a possible fungistatic effect of *Lp. plantarum* in fermented raw milk. In contrast, Luz et al. [38], studying whey fermented by *Lp. plantarum* 220 and 221, reported strong activity against mycotoxigenic fungi of the genus *Fusarium*, while only mild activity was observed against *Aspergillus* and *Penicillium*.

According to Table 6, before fermentation (T0), the percentage of positive samples for *Salmonella* spp. was 33.33% for both treatments. After 16 h of fermentation (TF), *Salmonella* spp. was detected only in T1. Compliance with Brazilian legislation—requiring absence of this microorganism in 25 g of product [39]—was achieved only at day 28 for both treatments (T1 and T2).

During fermentation and storage of T2, *Lp. plantarum* inhibited pathogenic and spoilage microorganisms. This antagonism is mainly attributed to growth competition and organic acid production; additionally, many lactic acid bacteria synthesize other antimicrobial compounds, such as bacteriocins, which enhance their preservative role [36].

## 4. Conclusions

In addition to exhibiting high viability, the presence of *Lactiplantibacillus plantarum* CNPC001 in co-culture with *Streptococcus thermophilus* QGE provided a superior preservative effect in fermented raw milk compared to that observed for the starter culture used alone, even with similar progressive reduction of pH and increase in acidity verified for both trials. The main bio-preservative effect of *Lp. plantarum* CNPC001 in co-culture with *S. thermophilus* was verified against *E. coli* and *Staphylococcus* spp. It should be emphasized that the present study has an exclusively scientific purpose, using raw milk solely due to its microbial diversity in order to better understand the bioconservation potential of *Lp. plantarum* CNPC001. Therefore, the data presented herein do not imply or suggest negligence regarding the adoption of good agricultural and/or food manufacturing practices, which must be ensured in the production of this type of product.

## Figures and Tables

**Table 1:** Ingredients used for the production of fermented raw milk T1 and T2.

Ingredients	Trial	
	T1	T2
Raw goat milk (g 100 g^−1^)	95.0	90.0
Native strain inoculum (g 100 g^−1^)	0	5.0
Starter culture inoculum (g 100 g^−1^)	5.0	5.0
Total (g 100 g^−1^)	100.0	100.0

T1 = trial 1 with *S. thermophilus* and T2 = trial 2 with *S. thermophilus* plus *Lp. plantarum*.

**Table 2:** Values of pH and titratable acidity (mean ± standard deviation) of the fermented raw milk treatments.

Trial	Time	pH	Titratable Acidity
T1	T0	6.39 ± 0.009 ^Db^	0.20 ± 0.02 ^Aa^
	TF	4.52 ± 0.036 ^Ca^	0.79 ± 0.07 ^Ba^
	D14	4.39 ± 0.017 ^Ba^	0.93 ± 0.01 ^Ca^
	D28	4.34 ± 0.010 ^Ab^	1.03 ± 0.03 ^Da^
T2	T0	6.35 ± 0.009 ^Da^	0.16 ± 0.04 ^Aa^
	TF	5.80 ± 0.009 ^Cb^	0.79 ± 0.04 ^Ba^
	D14	4.44 ± 0.015 ^Bb^	0.94 ± 0.03 ^Ca^
	D28	4.13 ± 0.019 ^Aa^	1.05 ± 0.02 ^Da^

^A, B, C,^ and ^D^ = different uppercase letters in the same column indicate significant differences (*p* ≤ 0.05) for the same trial among storage times. ^a^ and ^b^ = different lowercase letters in the same column indicate significant differences (*p* ≤ 0.05) among treatments on the same storage day. T1 = trial 1 with *S. thermophilus* and T2 = trial 2 with *S. thermophilus* plus *Lp. plantarum*. T0 = time before fermentation; TF = time after 16 h of fermentation; D14 = time after 14 days of storage; D28 = time after 28 days of storage.

**Table 3:** Viable populations of *Streptococcus thermophilus* in fermented goat dairy products during processing and storage at 4 ± 1 °C.

		Trial	
	Time	T1	T2
*S. thermophilus* (log CFU g^−1^)	T0	6.64 ± 0.06 ^Aa^	6.69 ± 0.08 ^Aa^
	TF	7.72 ± 0.03 ^Ba^	7.89 ± 0.07 ^Bb^
	D14	7.90 ± 0.01 ^Ca^	8.26 ± 0.18 ^Cb^
	D28	7.97 ± 0.02 ^Da^	8.61 ± 0.08 ^Db^

^A, B, C,^ and ^D^ = different uppercase letters in the same column indicate significant differences (*p* ≤ 0.05) for the same trial among storage times. ^a^ and ^b^ = different lowercase letters in the same row indicate significant differences (*p* ≤ 0.05) between treatments on the same storage day. T1 = trial 1 with *S. thermophilus* and T2 = trial 2 with *S. thermophilus* plus *Lp. plantarum.* T0 = time before fermentation; TF = time after 16 h of fermentation; D14 = time after 14 days of storage; D28 = time after 28 days of storage.

**Table 4:** Viable populations of *Lactiplantibacillus plantarum* in raw milk before fermentation (time 0), after fermentation (TF = 16 h), and during storage at 4 ± 1 °C (T2).

	Time	Trial
		T2
*Lactiplantibacillus plantarum* (log CFU g^−1^)	T0	6.43 ± 0.04 ^A^
	TF	6.61 ± 0.05 ^B^
	D14	7.99 ± 0.01 ^C^
	D28	8.60 ± 0.02 ^D^

^A, B, C,^ and ^D^ = different uppercase letters in the same column indicate significant differences (*p* ≤ 0.05) for the same trial among storage times. T2 = trial 2 with *S. thermophilus* plus *Lp. plantarum*. T0 = time before fermentation; TF = time after 16 h of fermentation; D14 = time after 14 days of storage; D28 = time after 28 days of storage. Source: research data.

**Table 5:** Populations of microbial contaminants (total coliforms, *E. coli*, *S. aureus*, and *Salmonella* spp.) detected in raw milk as well as raw milk supplemented with lactic cultures before fermentation (time 0), after fermentation (TF = 16 h), and during storage (14 and 28 days) at 4 ± 1 °C.

Contaminants	Trial	Bacterial Concentration (Minimum–Maximum Values, log CFU mL^−1^)					% Positive Samples * (No. Positive/Total Analyzed)	% Samples Above the Legal Limit ** (No. Above/Total Analyzed)	Maximum Tolerable Limit ***
		Raw Milk	T0	TF	D14	D28			
Coliforms	T1	4.97–8.00	4.25–4.70 ^Aa^	4.41–5.00 ^Aa^	3.09–3.90 ^Aa^	2.61–3.97 ^Aa^	100.0% (24/24) ^a^	100.0% (24/24) ^a^	2.0 log mL^−1^
	T2		3.00–4.58 ^Aa^	2.43–4.49 ^Aa^	1.26–2.78 ^Aa^	<1.00–1.96 ^Aa^	91.7% (22/24) ^a^	75.0% (18/24) ^a^	
*E. coli*	T1	2.90–3.58	3.43–3.52 ^Ba^	1.26–3.45 ^Aa^	<1.00 ^Aa^	<1.00 ^Aa^	50.0% (12/24) ^b^	50.0% (12/24) ^b^	1.0 log mL^−1^
	T2		3.48–3.53 ^Ba^	<1.00 ^Aa^	<1.00 ^Aa^	<1.00 ^Aa^	25.0% (6/24) ^a^	25.0% (6/24) ^a^	
*Staphylococcus* spp.	T1	3.18–3.77	3.15–4.51 ^Aa^	2.83–4.48 ^Ab^	2.08–3.26 ^Ab^	1.28–3.08 ^Ab^	100.0% (24/24) ^b^	-	-
	T2		2.99–3.72 ^Ba^	<2.00–2.30 ^Aa^	<2.00 ^Aa^	<2.00 ^Aa^	33.33% (8/24) ^a^	-	
Molds and yeasts	T1	3.40–3.81	3.46–3.62 ^Aa^	3.18–3.66 ^Aa^	3.62–3.96 ^Aa^	5.08–5.51 ^Aa^	100.0% (24/24) ^a^	100.0% (24/24) ^a^	3.0 log mL^−1^
	T2		3.26–3.60 ^Aa^	3.36–3.67 ^Aa^	3.34–3.52 ^Aa^	3.00–3.46 ^Aa^	100.0% (24/24) ^a^	95.8% (23/24) ^a^	

^A, B^ = different uppercase letters in the same row indicate significant differences (*p* ≤ 0.05) by the exact binomial test for the same trial across sampling periods, considering the null hypothesis (H_0_) that the number of positive samples is reduced to at least 33.33%. ^a, b^ = different lowercase letters in the same column indicate significant differences (*p* ≤ 0.05) by the exact binomial test for the same microorganism at the same sampling time or across the sampling periods (column * = total computed from T0 to D21), considering the null hypothesis (H_0_) that the number of positive samples or the number of samples above the legal limit (column **) is reduced to at least 33.33%. *** IN 46, Brazil (2007) for coliforms; IN 161, Brazil (2022) for *E. coli* and molds and yeasts. - = not available in current legislation. T1 = trial 1 with *S. thermophilus* and T2 = trial 2 with *S. thermophilus* plus *Lp. plantarum*. T0 = time before fermentation. TF = time after 16 h of fermentation. D14 = time after 14 days of storage. D28 = time after 28 days of storage.

**Table 6:** Presence of *Salmonella* spp. in raw milk as well as raw milk supplemented with lactic cultures before fermentation (time 0), after fermentation (TF =16 h), and during storage (14 and 28 days) at 4 ± 1 °C.

		Raw Milk	Sampling Periods				% Positive Samples ** (No. of Positives/Total Analyzed)	Maximum Tolerable Limit ***
			T0	TF	D14	D28		
% positive samples (no. positive/total analyzed)	T1	33.33% (2/6) *	33.33% (2/6) ^Ba^	33.33% (2/6) ^Bb^	33.33% (2/6) ^Ba^	0% (0/6) ^Aa^	25% (6/24) ^a^	Absent in 25 g
	T2		33.33% (2/6) ^Ba^	0% (0/6) ^Aa^	33.33% (2/6) ^Ba^	0% (0/6) ^Aa^	16.7% (4/24) ^a^	

^A, B^ = different uppercase letters in the same row indicate significant differences (*p* ≤ 0.05) by the exact binomial test for the same trial across sampling periods, considering the null hypothesis (H0) that the number of positive samples is reduced to 0%. ^a, b^ = different lowercase letters in the same column indicate significant differences (*p* ≤ 0.05) by the exact binomial test for *Salmonella* spp. at the same sampling time or across the sampling periods. * = for all trials, in a sampling day, the two positive plates were derived from the duplicate plate of the same enriched sample. ** = total computed from T0 to D21), considering the null hypothesis (H0) that the number of positive samples, or the number of samples above the maximum tolerable limit **, is reduced to 0%. *** maximum tolerable limit stablished by IN 161, Brazil (2022). T1 = trial 1 with *S. thermophilus*. T2 = trial 2 with *S. thermophilus* plus *Lp. plantarum*. T0 = time before fermentation. TF = time after 16 h of fermentation. D14 = time after 14 days of storage. D28 = time after 28 days of storage.

## Data Availability

The original contributions presented in the study are included in the article. Further inquiries can be directed to the corresponding author.
